# Audit of Prolactin Levels Monitoring for Inpatients on Antipsychotics in SABP

**DOI:** 10.1192/bjo.2022.464

**Published:** 2022-06-20

**Authors:** Zafrina Majid, Amit Fulmali, Bruce Tamilson, Ruth Bloxam, Deeksha Varma, Lubna Abdallah, Khalid Mirza

**Affiliations:** 1 Surrey & Borders NHS Foundation Trust, Surrey, United Kingdom; 2 East London NHS Foundation Trust, London, United Kingdom

## Abstract

**Aims:**

To establish whether our practice is meeting NICE and Maudsley guidelines in establishing baseline prolactin levels in an inpatient set-up before starting treatment with antipsychotic medications with a medium or high-risk of causing hyperprolactinaemia.

**Methods:**

Data were collected retrospectively from case notes of 127 patients from 9 wards at Surrey and Borders Partnership NHS Foundation Trust (SABP).

We reviewed if the baseline prolactin was measured for inpatients before commencing on antipsychotics with medium or high risk of hyperprolactinemia.

We reviewed if patients with elevated prolactin levels were assessed and managed appropriately.

**Results:**

SABP is currently achieving 43% in recording serum prolactin levels for inpatients who are on antipsychotics with medium or high-risk of hyperprolactinemia respectively.

Inpatient ward 76 patients out of total 127 were on antipsychotics with medium to high-risk of developing hyperprolactinemia.33 patients had their serum prolactin checked bringing the compliance to 43%,2 patients were excluded due to incomplete data bringing the sample size to 31.

3 had elevated prolactin. Out of 3 patients,1 patient was managed appropriately with MRI brain, followed by change of antipsychotic medication and repeat prolactin levels. For 1 patient, prolactin

level was rechecked. Unfortunately, no documentation of assessment of symptoms of hyperprolactinemia was found in all three patients case notes.

**Conclusion:**

The trust is falling short of meeting NICE and Maudsley guidelines of monitoring prolactin level. It is possible to introduce a robust system within the Trust so that we are complaint with a NICE and Maudsley prolactin monitoring guidelines.

We need to local Trust guidelines for management of hyperprolactinaemia in line with NICE and Maudsley guideline of monitoring prolactin levels.

Safety netting advice and leaflets explaining symptoms of hyperprolactinaemia should be provided to all the patients on antipsychotics with medium to high risk of developing hyperprolactinemia.

